# Development and validation of a novel tool for identification and categorization of non-technical errors associated with surgical mortality

**DOI:** 10.1093/bjs/znae253

**Published:** 2024-10-18

**Authors:** Jesse D Ey, Victoria Kollias, Matheesha B Herath, Octavia Lee, Martin H Bruening, Adam J Wells, Guy J Maddern

**Affiliations:** Department of Surgery, University of Adelaide, The Queen Elizabeth Hospital, Woodville, South Australia, Australia; Department of Surgery, University of Adelaide, The Queen Elizabeth Hospital, Woodville, South Australia, Australia; Department of Surgery, University of Adelaide, The Queen Elizabeth Hospital, Woodville, South Australia, Australia; Department of Surgery, University of Adelaide, The Queen Elizabeth Hospital, Woodville, South Australia, Australia; Department of Surgery, University of Adelaide, The Queen Elizabeth Hospital, Woodville, South Australia, Australia; Department of Neurosurgery, Royal Adelaide Hospital, Adelaide, South Australia, Australia; Department of Surgery, University of Adelaide, The Queen Elizabeth Hospital, Woodville, South Australia, Australia

## Abstract

**Background:**

Up to half of all surgical adverse events are due to non-technical errors, making non-technical skill assessment and improvement a priority. No specific tools are available to retrospectively identify non-technical errors that have occurred in surgical patient care. This original study aimed to develop and provide evidence of validity and inter-rater reliability for the System for Identification and Categorization of Non-technical Error in Surgical Settings (SICNESS).

**Methods:**

A literature review, modified Delphi process, and two pilot phases were used to develop and test the SICNESS tool. For each pilot, 12 months of surgical mortality data from the Australian and New Zealand Audit of Surgical Mortality were assessed by two independent reviewers using the SICNESS tool. Main outcomes included tool validation through modified Delphi consensus, and inter-rater reliability for: non-technical error identification and non-technical error categorization using Cohen’s κ coefficient, and overall agreement using Fleiss’ κ coefficient.

**Results:**

Version 1 of the SICNESS was used for pilot 1, including 412 mortality cases, and identified and categorized non-technical errors with strong–moderate inter-rater reliability. Non-technical error exemplars were created and validated through Delphi consensus, and a novel mental model was developed. Pilot 2 included an additional 432 mortality cases. Inter-rater reliability was near perfect for leadership (κ 0.92, 95% c.i. 0.82 to 1.00); strong for non-technical error identification (κ 0.89, 0.84 to 0.93), communication and teamwork (κ 0.89, 0.79 to 0.99), and decision-making (κ 0.85, 0.79 to 0.92); and moderate for situational awareness (κ 0.79, 0.71 to 0.87) and overall agreement (κ 0.69, 0.66 to 0.73).

**Conclusion:**

The SICNESS is a reliable and valid tool, enabling retrospective identification and categorization of non-technical errors associated with death, occurring in real surgical patient interactions.

## Teaser

This study documented the development of the System for Identification and Categorization of Non-technical Error in Surgical Settings. This is the first tool to enable retrospective identification and categorization of non-technical errors occurring in real-world surgical patient care. The results of this study demonstrated satisfactory reliability and validity of this novel tool.

## Introduction

Non-technical skills (NTS), the cognitive and interpersonal components of surgical professionalism, underpin every aspect of modern surgical practice^1^. With over 310 million surgical procedures performed globally each year^2^, the incidence of adverse events is 14.4%, of which 24–51% are due to failings of NTS. Failures of NTS contribute significantly to the burden of preventable deaths across multiple surgical specialties and clinical contexts, including non-operative, perioperative, and intraoperative environments^3–6^. As such, assessing and improving NTS has become a priority for surgical training organizations^7–11^.

Over 70 assessment tools for NTS exist. They are heterogeneous in design, content, and scoring scales; however, one characteristic is consistent: they are observational in nature^12–14^. To use these tools, assessors must view surgical interactions in real time or by reviewing prospectively recorded clinical encounters prospectively. Owing to this, NTS assessment activities have been restricted to small cohorts, routine surgical situations, or low-stakes simulated environments planned for assessment ahead of time^14^. Adverse events are unintended, unpredictable, and more likely to occur in situations with high-pressure emergency components^8^. Therefore, the circumstances in which adverse events occur are poorly represented in NTS assessment activities. A proposed method to incorporate these circumstances is retrospective audit of real surgeon–patient encounters using patient medical records. A tool enabling identification of non-technical errors through retrospective assessment of medical records would be an invaluable adjunct to clinical audit processes, ubiquitous in modern surgery, thereby providing insight into NTS shortcomings at individual, departmental, and systems levels^15,16^. This in turn would provide targets for root cause analysis to direct future interventions and ultimately prevent future adverse events.

The aims of this study were to develop the System for Identification and Categorization of Non-technical Error in Surgical Settings (SICNESS), and provide evidence of validity and inter-rater reliability.

## Methods

This study used a multistep design including literature review, modified Delphi process, and two pilot phases conducted over 12 months (April 2023 to April 2024). Pilot phases used surgical mortality data from the Australian and New Zealand Audit of Surgical Mortality (ANZASM) for two time intervals: January to December 2012 (pilot 1) and January to December 2019 (pilot 2). Intervals separated by several years were chosen to reduce time-period bias. The pilot 1 cohort represents a historical cohort recent enough to be relevant to modern surgery, whereas the pilot 2 cohort represents the most recent cohort available not influenced by the COVID-19 pandemic. ANZASM is a national, peer-reviewed surgical audit overseen by the Royal Australasian College of Surgeons (RACS). All surgical patient deaths in Australia (excluding New South Wales) are reported to ANZASM, including any patients with whom a surgeon had significant involvement, even if they did not undergo a surgical procedure. The responsible surgeon provides a detailed report regarding patient care, which is then deidentified and sent for external peer review. This report is assessed by an independent consultant surgeon and, if required, by a second independent consultant surgeon with access to patient medical records for the purposes of identifying aspects of patient management that could be improved or may have contributed to patient death. These aspects are classified into three levels of seriousness: area for consideration, area of concern, or adverse event^17^. For both pilot intervals, all patient deaths, flagged as having an area of concern or adverse event, were included with no other exclusions. This project was approved by the RACS ethics committee.

## System development

A literature review was conducted across three peer-reviewed databases (Embase, MEDLINE, PubMed). Studies reporting NTS assessment tool design, validation, or use were reviewed. A modified Delphi process was used to inform tool design and refinement. Modified Delphi experts included three consultant general surgeons (G.J.M., M.H.B., A. Anthony), one consultant orthopaedic surgeon (J. North), and a consultant anaesthetist (D. Sainsbury), all with extensive experience in human-factors research and NTS assessment at a national level. Delphi rounds were anonymous, with consensus defined as 75% agreement between experts, as described previously^18^. Failure to reach consensus resulted in discussion and, if stability persisted, agreement would be determined by majority vote.

## Pilot 1

The SICNESS was applied to 12 months of deidentified ANZASM assessment summaries (January to December 2012) by two independent reviewers: one a researcher (J.D.E.) and the other a surgical registrar (V.K.), both with expertise in surgical NTS attained through previous research, RACS-approved NTS course material, and direct teaching from an expert RACS faculty. Inter-rater reliability was assessed for three outcomes: whether there was an identifiable non-technical error; if so, which NTS domain the error belonged to; and overall agreement.

## Tool refinement

Errors identified by both reviewers during pilot 1 were analysed thematically and grouped into possible non-technical error exemplars. Exemplars were distributed to modified Delphi experts to establish whether the errors represented failings of surgical NTS, and which NTS domain individual exemplars belong to. Once consensus had been reached, exemplars were included in the refined SICNESS.

## Pilot 2

The refined SICNESS was applied to an additional 12 months of deidentified ANZASM case summaries (January to December 2019) by two independent reviewers (J.D.E., M.B.H.) with experience identical to that of pilot 1. Inter-rater reliability was assessed for the same outcomes as in pilot 1.

## Statistical analysis

Inter-rater reliability was assessed using Cohen’s κ coefficient for the following outcomes. Is there a non-technical error identifiable (yes/no)? If there is an error, to which category does it belong—decision-making, situational awareness, communication/teamwork, or leadership (4 variables)? The inter-rater reliability for overall agreement (patients for whom reviewers agreed on non-technical error, categorization of error, and number of domains involved) was assessed using Fleiss’ κ coefficient. Interpretation of Cohen’s and Fleiss κ coefficients was: 0–0.20, none; 0.21–0.39, minimal; 0.40–0.59, weak; 0.60–0.79, moderate; 0.80–0.90, strong; and more than 0.90, almost perfect^19^.

## Results

### System development

Literature review identified 75 NTS assessment tools, 14 of which were relevant to surgeon NTS, with heterogeneity in number of NTS domains included and nomenclature used^20–33^. All tools assessed NTS by scoring observable behaviours using Likert scales. None were designed to identify non-technical errors retrospectively using medical records, and none measured NTS in the context of surgical morbidity or mortality.

**Fig. 1:**
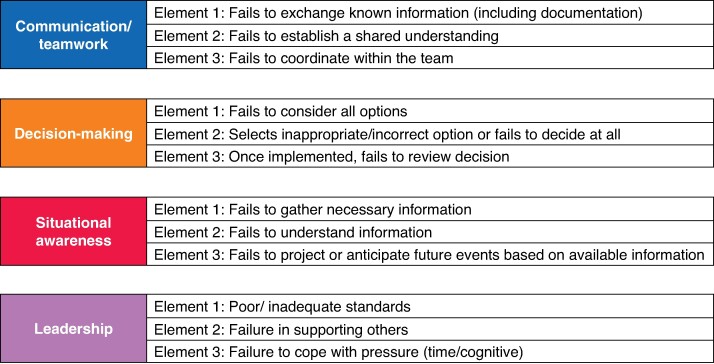
Consensus agreed domains and elements of SICNESS SICNESS, System for Identification and Categorization of Non-technical Error in Surgical Settings.

Through Delphi consensus, the Non-Technical Skills for Surgeons (NOTSS) tool was determined to be a satisfactory tool for adaptation^33^. This tool includes four NTS domains: communication/teamwork, decision-making, situational awareness, and leadership. Each domain is further divided into elements, representing the behaviours required for a surgeon to demonstrate satisfactory NTS for each domain^1^. The purpose of the SICNESS is to identify situations where NTS have not been satisfactory, so the NOTSS elements were adapted to represent failings of NTS, or ‘errors’ (*Fig. 1*) Likert scale scoring was replaced with binary and categorical scoring for the following two variables. Is there an identifiable non-technical error (yes/no)? If yes, which domain is the non-technical error representative of?

### Pilot 1

Some 412 cases were assessed. Strong inter-rater reliability was demonstrated for non-technical error, communication/teamwork, situational awareness, and leadership. Moderate inter-rater reliability was shown for decision-making and overall agreement across the four domains (*Table 1*). Overall agreement was achieved in 79.2% of cases.

**Table 1:** Pilot 1 inter-reviewer reliability for three measured outcomes (6 variables)

	Simple κ	Interpretation
Identification of non-technical error	0.83 (0.77, 0.89)	Strong agreement
Communication and teamwork	0.82 (0.74, 0.89)	Strong agreement
Decision-making	0.80 (0.72, 0.87)	Moderate agreement
Situational awareness	0.89 (0.83, 0.94)	Strong agreement
Leadership	0.82 (0.73, 0.92)	Strong agreement
Overall agreement (4 domains)	0.64 (0.61, 0.67)*	Moderate agreement

Values in parentheses are 95% confidence intervals. *Weighted Fleiss’ κ coefficient.

### System refinement

In pilot 1, non-technical errors were identified by both reviewers in 285 cases, providing 570 error reports. Thematic analysis yielded 50 distinct non-technical error exemplars across NTS domains: communication/teamwork (15), decision-making (13), situational awareness (12), and leadership (10). Exemplars were distributed to Delphi experts for assessment. Consensus was achieved for all 50 exemplars, which were subsequently included in the SICNESS (*Table S1*).

**Fig. 2:**
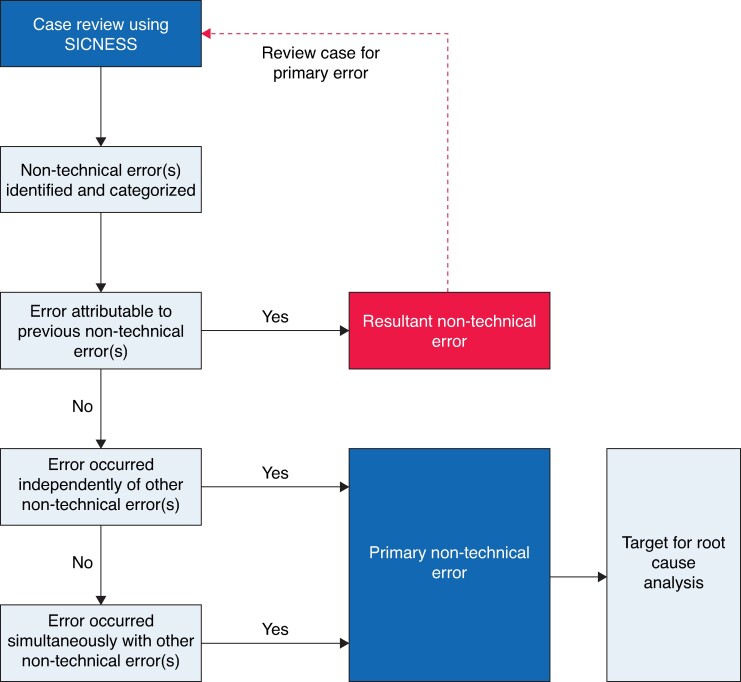
Novel mental model: primary *versus* resultant non-technical errors SICNESS, System for Identification and Categorization of Non-technical Error in Surgical Settings.

Poorer overall agreement between reviewers was demonstrated for cases with more than one non-technical error identified. This stimulated the development of a novel mental model to aid categorization of non-technical errors. The mental model enables categorization of errors into primary non-technical error (those occurring independently of other non-technical errors) or resultant non-technical errors (those resulting from the misunderstanding or misinformation caused by a preceding non-technical error) (*Fig. 2*).

### Pilot 2

Using the refined SICNESS, 430 additional mortality cases were assessed. Inter-rater reliability was almost perfect for leadership; strong for non-technical error, communication/teamwork, and decision-making; and moderate for situational awareness and overall agreement across all four domains. (*Table 2*) Improvement in inter-rater reliability was demonstrated for five of six variables: non-technical error (κ 0.83 and 0.89 for pilots 1 and 2 respectively), communication/teamwork (κ 0.82 and 0.89), decision-making (κ 0.80 and 0.85), leadership (κ 0.82 and 0.92), and overall (κ 0.64 and 0.69). Decreased inter-rater reliability for situational awareness was demonstrated (κ 0.89 and 0.79). Overall agreement was achieved for 84.9% of cases.

**Table 2:** Pilot 2 inter-reviewer reliability for three measured outcomes (6 variables)

Variable	Simple κ	Interpretation
Identification of non-technical error	0.89 (0.84, 0.93)	Strong agreement
Communication and teamwork	0.89 (0.79, 1.00)	Strong agreement
Decision-making	0.85 (0.79, 0.92)	Strong agreement
Situational awareness	0.79 (0.71, 0.87)	Moderate agreement
Leadership	0.92 (0.82, 1.00)	Almost-perfect agreement
Overall agreement (4 domains)	0.69 (0.66, 0.73)*	Moderate agreement

Values in parentheses are 95% confidence intervals. *Weighted Fleiss’ κ coefficient.

### Validity

Face and content validity were demonstrated for domains, elements, and exemplars, achieved through modified Delphi consensus. Construct validity of the included NTS domains has been demonstrated previously^34^.

## Discussion

The purpose of this study was to develop a novel tool enabling identification and categorization of non-technical errors using existing surgical patient data to guide future improvement strategies. Using the final version of the SICNESS, non-technical errors could be identified with strong inter-rater reliability and categorized into NTS domains with near-perfect to moderate inter-rater reliability. The addition of specific, validated exemplars improved inter-rater reliability for five of six measured outcomes. Inter-rater reliability for situational awareness decreased from strong to moderate, an unexpected result reflecting the complexity of non-technical error categorization. However, despite this, overall agreement between reviewers increased from 79.2 to 84.9%, demonstrating clear improvement.

The novel mental model developed in this study allows categorization of identified non-technical errors into two types: primary and resultant. Primary non-technical errors provide a specific target for root cause analysis and future preventative strategies^35^. Once a primary non-technical error has occurred, the cognitive landscape is altered, and misinformation often leads to further errors. These resultant errors occur as a consequence of the preceding primary error, and so are unlikely to be effective targets for future preventative strategies.

An example of this is as follows. A junior team member fails to recognize necrotizing fasciitis, mistaking the diagnosis as cellulitis—a situational awareness error. Owing to the misdiagnosis, the junior team member does not communicate with the consultant surgeon urgently—a communication error. The result is delayed appreciation of the situation by the consultant, leading to delayed surgical debridement, patient deterioration, and death. There are two non-technical errors identified in this example; however, the communication error was caused by the initial failure of the junior team member to recognize necrotizing fasciitis. Therefore, the situational awareness error was the primary error, and the subsequent communication error was a resultant error. In this example, the institution of a communication workshop would be unlikely to prevent the same error from recurring when evidently the primary error was a failure to recognize a surgical emergency. Further examples with explanations are provided in the SICNESS User Manual available in *Supplementary material*.

Previous studies^8,9^ investigating NTS and adverse events have included small, non-generalizable cohorts, or included only components of NTS, such as communication, rather than all NTS domains. This has resulted in incomplete understanding of the incidence and characteristics of non-technical errors occurring in the wider surgical community. Furthermore, all pre-existing NTS assessment tools are designed for prospective surgeon NTS assessment, but none in the context of patient morbidity or mortality. Owing to these limitations, NTS improvement activities, though beneficial for surgeon-centric outcomes, such as improved self-efficacy, self-confidence, and technical skill, have been largely ineffective in improving patient outcomes^22,36–38^. The SICNESS was developed to overcome these limitations by using a large patient population, representative of 15 surgical specialties, and multiple clinical contexts, including intraoperative, perioperative, and non-operative settings^39,40^. The SICNESS allows comprehensive assessment inclusive of all surgical NTS domains, and is the first tool to enable retrospective identification of non-technical errors associated with patient harm in real-world surgical patients.

Retrospective analysis for adverse events already occurs ubiquitously in surgery for quality improvement purposes in the form of clinical audits, although not specifically for non-technical components^15,16^. Although some audit processes do include components of NTS, these are primarily surgeon-focused and neither comprehensive nor standardized^41^. The SICNESS tool can be readily introduced into existing audit practices, providing a comprehensive and standardized tool enabling systemic use with minimal increased resource expenditure.

The SICNESS was developed for use with patient medical records, a resource universally available in modern surgery. Medical records are contributed to by multiple team members, at multiple time points, and include pertinent information about individual, departmental, and hospital-level practices, enabling comprehensive non-technical error assessment^42^. However, medical records are not perfect: incorrect information is often recorded and important details omitted^43,44^. It is these imperfections that often herald identification of non-technical errors. Errors and omissions in medical records represent a failure to share known information—a communication/teamwork non-technical error. Using the SICNESS, these errors could be considered in context of the available medical record information and further scrutinized to discern whether they are primary or resultant non-technical errors enabling relatively comprehensive assessment regardless.

Previous studies^45–48^ used surgeon recall through interview processes as an alternative to medical record review for retrospective analysis of non-technical error^8^. Memory is prone to a multitude of recall biases, providing only the perspective of the interviewee, and such data capture is financially and logistically prohibitive on a large scale.

There are limitations to the present study. The SICNESS was piloted using surgical mortality cases already summarized by consultant surgeons. To use the tool in real-world settings, the assessor must be able to formulate their own summary from patient medical records. The level of comprehension required to do so is no greater than that required to conduct standard clinical audits, a task frequently conducted by junior team members^49^. However, further studies to demonstrate usability by junior team members are needed. Next, reviewers used the SICNESS to identify non-technical errors associated with patient death, yet this demographic represents only a proportion of surgical patients. To be widely generalizable, the tool must be able to identify non-technical errors associated with patient morbidity. It is likely that this tool can identify non-technical errors associated with less severe endpoints; however, further studies are required to confirm this. Finally, although the SICNESS can provide targets for future improvement interventions through the reliable identification and categorization of non-technical errors, the efficacy of any resulting interventions is beyond the scope of this study and future investigations are required to define this.

The SICNESS is reliable, generalizable, and readily adoptable for quality improvement purposes. This tool should be considered for integration into national or system-level audits to standardize non-technical error assessment, and quantify the incidence and impact of non-technical errors in surgery. It can be introduced at a local level as an adjunct to morbidity and mortality conferences and other quality improvement processes to identify common and severe non-technical errors, guide improvement activities, and reduce preventable adverse events.

## Supplementary Material

znae253_Supplementary_Data

## Data Availability

Study data are available on request to the corresponding author.
