# Supplementary material for: Development and validation of a novel tool for identification and categorization of non-technical errors associated with surgical mortality
Source: Br J Surg. 2024 Oct 18;111(10):znae253. doi: 10.1093/bjs/znae253 (PMC11488386; doi:10.1093/bjs/znae253)
Supplement: znae253_Supplementary_Data [file znae253_supplementary_data.docx]

**Title: Development and validation of a novel tool for identification and categorisation of non-technical errors associated with surgical mortality.**

Authors: Jesse D Ey^1^ (BMedSc Hons), Victoria Kollias^1^ (MBBS), Matheesha B Herath^1^(MBBS), Octavia Lee^1^ (BMedSc), Martin H Bruening^1^ (MS), Adam J Wells^2^ (PhD), Guy J Maddern^1^ (PhD)

^1^Affiliation: Department of Surgery, The University of Adelaide, The Queen Elizabeth Hospital, 28 Woodville Road, Woodville, SA 5011, Australia.

^2^Affiliation : Department of Neurosurgery, The Royal Adelaide Hospital, Port Road, Adelaide, SA 5000, Australia.

**Corresponding author.** Professor Guy J. Maddern, Department of Surgery, The University of Adelaide, The Queen Elizabeth Hospital, 28 Woodville Road, Woodville, SA 5011, Australia.

Orcid ID: 0000-0003-2064-181X

**Supplementary Materials - Index**

| **Supplementary Appendixes** |  |
| --- | --- |
| SICNESS User Manual | *page 2-3* |
| **Supplementary Figures and Tables** |  |
| Table 1: Non-technical error exemplars | *page 4-7* |
|  |  |

**Supplementary Appendixes**

User manual for the **S**ystem for **I**dentification and **C**ategorisation of **N**on-technical **E**rror in **S**urgical **S**ettings (SICNESS).

**Scope:**

- To critically evaluate a clinical scenario and determine if a Non-technical error has contributed to, or may contribute to, an adverse healthcare outcome.
- This tool is intended to be used to guide root cause analysis and inform future error preventative strategies.

**How to use the SICNESS**

1. Read the available patient case notes to formulate a summary of the patient care journey. Critically assess the patient care journey to identify any components of care that deviate away from best practice including but not limited to errors of care, omissions of care or investigation, or concerning events.
2. Do these identified areas of concern fit into one of the non-technical error exemplars within the SICNESS tool? (Supplementary Table 1) NOTE: the exemplars are not exhaustive, if the area of concern does not fit neatly into one of the exemplars, consider if it fits into one of the non-technical error elements, or domains.
3. If yes, you have identified a non-technical skill error – apply the mental model of primary vs resultant error outlined below. (Figure 2 in main manuscript)
   1. Is this identified non-technical skill error likely caused by another identifiable error?
      1. If yes, this is a resultant error. This means another non-technical error has occurred, continue to assess the case to identify the primary error.
      2. If no, this is a primary error. Include this error in your assessment and characterise the element, and domain it most appropriately belongs to using the exemplars and elements within the SICNESS tool.
      3. If the error is intimately connected with another non-technical error or errors occurring at the same time point, all these errors can be considered primary. Include this error in your assessment and characterise the element, and domain it most appropriately belongs to using the exemplars and elements within the SICNESS tool

NOTE: there can be more than one primary error per case and there may be multiple resultant errors for each primary error. Please see below for examples.


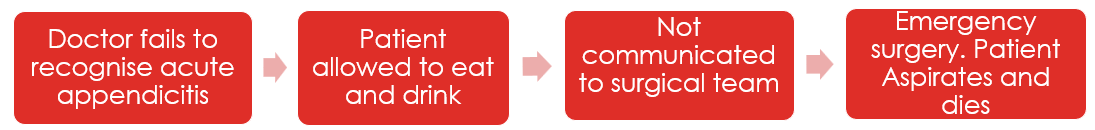
**Example 1:** Primary vs resultant error

**Example 1 explanation:** In this example case, a patient presents to the emergency department with abdominal pain, vomiting, and diarrhoea. The patient has appendicitis. The admitting doctor doesn’t recognise the clinical stigmata of appendicitis and diagnoses the patient with gastroenteritis – this is a situational awareness error. As the patient has been misdiagnosed, the admitting doctor allows the patient to eat and drink as they please – this is a decision-making error. Later, the patient’s appendix ruptures resulting in emergency surgery. The admitting doctor did not communicate that the patient was not fasted because they never anticipated the patient would require surgery – this is a communication/ teamwork error. In the theatre, upon anaesthetic induction, the patient aspirates and dies. There are three identified non-technical skill errors in this case, however, the decision-making error and subsequent communication error were likely caused by the initial failure of the admitting doctor to recognise appendicitis, therefore the situational awareness error was a primary error and should undergo root cause analysis, while the subsequent communication and decision-making errors are resultant.

**Example 2**: Simultaneous primary non-technical error

**
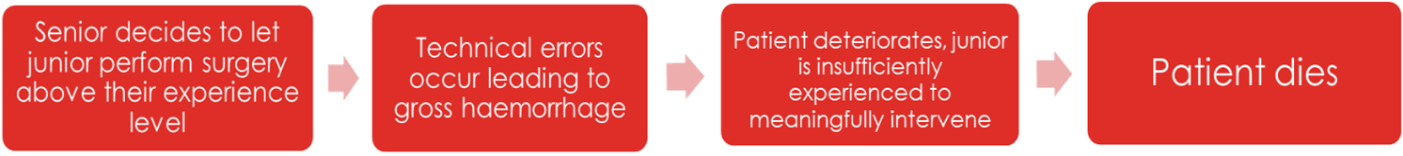
**

**Example 2 explanation**: In this case, the consultant surgeon has made the decision to put a junior team member in an inappropriate situation by letting them operate when not sufficiently experienced and without the appropriate support that the clinical context would require. These are errors in both leadership and decision making. However, it is not possible to determine if poor leadership led to a decision-making error, or the decision-making error makes the surgeon a poor leader. Rather than guessing and introducing subjective bias, instead, report both errors as primary errors so that formal root cause analysis can be conducted.


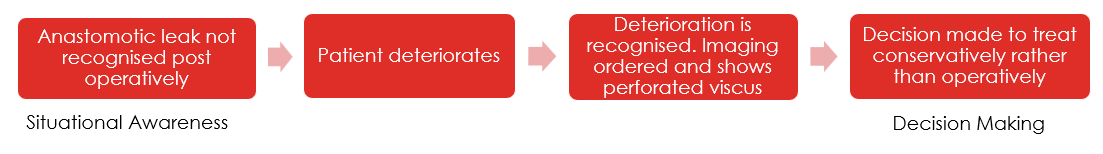
**Example 3:** Multiple primary non-technical errors

**Explanation 3 explanation:** In this case, a patient undergoes surgery including anastomosis. In the days following surgery, the patient develops stigmata of an anastomotic leak which is not recognised by the surgical team. Patient deteriorates further at which point the clinical decline is appreciated. Appropriate imaging is ordered and free gas under the diaphragm is seen, representing the leak. The correct clinical decision is to take the patient back to theatre but in this case, the surgeon makes the decision to treat conservatively. The result of which is further deterioration and patient death. This case has two identifiable non-technical errors. The failure to recognise clinical stigmata of an anastomotic leak represents a situational awareness error. Once deterioration was identified the correct imaging was ordered and the correct diagnosis was made, yet the decision to treat conservatively was a decision-making error. The initial situational awareness error did not influence the subsequent decision-making error as the at the time of the decision, the surgeon had all the necessary information. This case represents two independent non-technical errors occurring and both should undergo root cause analysis.

**Supplementary Figures and Tables**

| **Domain** | **Element** | **Exemplar** |
| --- | --- | --- |
| **Communication & Teamwork** | Fails to exchange known information | - Surgical team not informed about patient deterioration - Surgical team not informed about pre-existing medical conditions or severity of comorbidity - Surgical team not informed about patient readmission soon after operation - Surgical team fails to inform other team/team members about patient transfer - Surgical team fails to inform other team/team members about patient deterioration - Junior team member fails to escalate care to senior team member - Junior team member fails to provide accurate/complete/timely information to senior team member - Junior team member fails to seek advice/assistance from senior team member - Surgical team member fails to provide accurate/complete/timely information to appropriate medical team(s) - Patient consent incomplete or inappropriate due to cognitive impairment/incompetence/failure to secure translator - Completed surgical management plan did not match proposed/ agreed upon management plan for which consent was sought. - Missing/incomplete/inaccurate documentation resulting in inappropriate medical management - Missing/incomplete/inaccurate documentation resulting in misunderstanding of clinical picture - Disagreement between team/team members leading to delay in diagnosis/ investigation/ treatment - Surgical team/ team member not informed about relevant changes to patient medication or circumstances |
|  | Fails to establish a shared understanding |  |
|  | Fails to coordinate within the team or across other teams |  |
| Domain | Element | Exemplar |
| **Decision Making** | Fails to consider all options | - Surgeon offers/performs futile surgery - Surgeon operates despite presence of known physiological derangement - Patient provided inappropriately conservative management for the clinical context- non-operative or operative - Patient provided inappropriately aggressive surgical management for the clinical context - Surgical procedure includes additional elective components not necessary to achieve primary goal of procedure - Patient prescribed incorrect/inappropriate DVT prophylaxis as per clinical guidelines for the clinical context- including no DVT prophylaxis - Patient prescribed contraindicated medications - Patient prescribed inappropriate/incorrect medications for known clinical context - Surgery performed on patient known to be unstable or unwell despite procedure being unlikely to reverse instability, or illness - Surgical team/team member fails to initiate investigation/intervention appropriate for the identified clinical context - Surgeon encounters difficult pathology/ difficulty anatomy intraoperatively but fails to consider alternative course of action appropriate for clinical context - Team member aware of medical deterioration/ deranged physiology warranting consultation with another team yet does not consult with other team. - Surgeon proceeds with operation/ intervention at inappropriate time despite being aware of increased risk factors |
|  | Selects inappropriate or incorrect option |  |
|  | Once decision implemented, fails to review decision |  |
| Domain | Element | Exemplar |
| **Situational Awareness** | Fails to gather information | - Team member fails to complete appropriately comprehensive preoperative assessment - Team member misinterprets apparent clinical signs and symptoms - Team member fails to recognise clinical signs and symptoms - Team member fails to appreciate the need for investigation/ tests/ interventions appropriate for the clinical context - Team member misinterprets the results of ordered blood tests/imaging/ other investigations - Team member fails to appreciate the severity of patient illness leading to delay or omission of treatment - Team member fails to appreciate the impact of delaying investigation/ intervention/ treatment - When presented with an unexpected complication intraoperatively, team member fails to appreciate impact on predetermined management plans. - Team member fails to review pertinent documentation required for best patient care. - Team member misses diagnosis despite clinical evidence expected to be recognised by a person of their experience level - Patient incorrectly diagnosed due to misinterpretation of the clinical context by team member - Team member failed to understand/ or missed patient signs/symptoms that would reasonably warrant consultation with another team |
|  | Fails to understand information |  |
|  | Fails to predict or anticipate future events |  |
| Domain | Element | Exemplar |
| **Leadership** | Poor/Inadequate standards | - Senior/consultant not involved in patient pre-operative care to a degree appropriate for the clinical context - Senior/consultant not involved in patient intra-operative care to a degree appropriate for the clinical context - Senior/consultant not involved in patient post-operative/follow-up care to a degree appropriate for the clinical context - Senior/consultant fails to respond to reasonable request for assistance or advice from junior team members - Senior/consultant fails to respond to reasonable request for assistance or advice from non-immediate team members including nursing, anaesthetics, and consulting medical teams - Action or inaction of senior team member resulting in junior team member in an inappropriate position - Action or inaction of senior team member resulting in junior team member making decision outside of their scope of expertise - Senior/ consultant not contactable or available when it is expected that they should be - Senior/consultant aware of situation yet fails to intervene in a manner appropriate for the clinical context - Senior/consultant fails to complete tasks to an appropriate standard expected of their expertise level |
|  | Fails to support others |  |
|  | Fails to cope with pressure (time, stress, cognitive) |  |

Table 1: Non-technical error exemplars
